# A continuity of care programme for women at risk of preterm birth in the UK: Process evaluation of a hybrid randomised controlled pilot trial

**DOI:** 10.1371/journal.pone.0279695

**Published:** 2023-01-12

**Authors:** Cristina Fernandez Turienzo, Louise H. Hull, Kirstie Coxon, Mary Bollard, Pauline Cross, Paul T. Seed, Andrew H. Shennan, Jane Sandall, on behalf of the POPPIE Pilot Collaborative Group

**Affiliations:** 1 Faculty of Life Sciences & Medicine, Department of Women and Children’s Health, King’s College London, London, United Kingdom; 2 Department of Health Services and Population Research, Centre for Implementation Science, Institute of Psychiatry, Psychology & Neuroscience, King’s College London, London, United Kingdom; 3 Department of Midwifery, Kingston University and St. George’s, University of London, United Kingdom; 4 Maternity Services, Lewisham and Greenwich NHS Trust, London, United Kingdom; 5 Department of Public Health, London Borough of Lewisham, London, United Kingdom; Wayne State University, UNITED STATES

## Abstract

**Background:**

The development and evaluation of specific maternity care packages designed to address preterm birth remains a public health priority. We aim to evaluate the implementation, context, and potential mechanisms of action, of a new care pathway that combined midwifery continuity of care with a specialist obstetric clinic for women at risk of preterm birth (POPPIE) in London (UK).

**Methods:**

We did a multiphase mixed method triangulation evaluation nested within a hybrid type 2, randomised controlled trial in London (United Kingdom). Pregnant women with identified risk factors for preterm birth were eligible for trial participation and randomly assigned (1:1) to either midwifery continuity of care linked to a specialist obstetric clinic (POPPIE group) or standard maternity care. The primary outcome was a composite of appropriate and timely interventions for the prevention and/or management of preterm labour and birth, analysed according to intention to treat. Clinical and process outcome data were abstracted from medical records and electronic data systems, and coded by study team members, who were masked to study group allocation. Implementation data were collected from meeting records and key documents, postnatal surveys (n = 164), semi-structured interviews with women (n = 30), healthcare providers and stakeholders (n = 24) pre-, mid and post implementation. Qualitative and quantitative data from meeting records and key documents were examined narratively. Qualitative data from interviews were analysed using three thematic frameworks: Proctor’s (for implementation outcomes: appropriateness, adoption, feasibility, acceptability, fidelity, penetration, sustainability), the Consolidated Framework for Implementation Research (for determinants of implementation), and published program theories of continuity models (for potential mechanisms). Data triangulation followed a convergent parallel and pragmatic approach which brought quantitative and qualitative data together at the interpretation stage. We averaged individual implementation measures across all domains to give a single composite implementation strength score which was compared to the primary outcome.

**Results:**

Between May 9, 2017, and Sep 30, 2018, 553 women were assessed for eligibility and 334 were enrolled with less than 6% of loss to follow up (169 were assigned to the POPPIE group; 165 were to the standard group). There was no difference in the primary outcome (POPPIE group 83·3% versus standard group 84·7%; risk ratio 0·98 [95% CI 0·90 to 1·08]). Appropriateness and adoption: The introduction of the POPPIE model was perceived as a positive fundamental change for local maternity services. Partnership working and additional funding were crucial for adoption. Fidelity: More than 75% of antenatal and postnatal visits were provided by a named or partner midwife, and a POPPIE midwife was present in more than 80% of births. Acceptability: Nearly 98% of women who responded to the postnatal survey were very satisfied with POPPIE model. Quantitative fidelity and acceptability results were supported by the qualitative findings. Penetration and sustainability: Despite delays (likely associated with lack of existing continuity models at the hospital), the model was embedded within established services and a joint decision was made to sustain and adapt the model after the trial (strongly facilitated by national maternal policy on continuity pathways). Potential mechanisms of impact identified included e.g. access to care, advocacy and perceptions of safety and trust. There was no association between implementation measures and the primary outcome.

**Conclusions:**

The POPPIE model of care was a feasible and acceptable model of care that was implemented with high fidelity and sustained in maternity services. Larger powered trials are feasible and needed in other settings, to evaluate the impact and implementation of continuity programmes in other communities affected by preterm birth and women who experience social disadvantage and vulnerability.

**Trial registration:**

UKCRN Portfolio Database (prospectively registered, 24 April 2017): 31951.

ISRCTN registry (retrospectively registered, 21 August 2017): ISRCTN37733900.

## Introduction

Preterm birth (PTB, birth at < 37 weeks’ gestation) remains a crucial issue in improving outcomes and quality of maternal, newborn and child health care. One in ten babies worldwide are born too soon and over a million die from complications associated to their prematurity [1]. Many of those who survive can face adverse lifelong health consequences which impact on families, societies, and health systems [2]. Despite efforts to reduce the prevalence, improve clinical management and decrease neonatal mortality and morbidity, PTBs continue to rise in most countries [3]. Although most PTBs are spontaneous and only a small proportion are provider-initiated due to maternal or fetal reasons, the cause is still unknown in up to half of the cases and involves multiple and overlapping factors (e.g. infections, chronic diseases, obstetric history such as previous PTB, smoking, psychosocial stress, domestic violence, social deprivation) [4]. Thus, achievement of public health strategies to prevent PTBs has been challenging and developing and evaluating specific maternity packages of care, that incorporate single effective interventions designed to address prematurity, remains a public health priority [5,6]. Models of midwifery continuity of care have been the only health system intervention shown to reduce PTB and increase perinatal survival, but no evidence exists for women with risk factors for PTB [7,8].

Complex interventions, such as continuity models and other maternity care packages, are composed of multiple and different interacting components, and non-linear causal pathways [9]. Thus, their impact is dependent on the degree to which an intervention is implemented as intended, in specific contexts and population groups. A clear understanding of the most appropriate strategies to deliver them and how they may work and be adapted in varying contexts is vital [10]. Randomised controlled trials (RCTs) are the most rigorous way to evaluate the clinical impact of interventions, regardless of their complexity, but are often criticised for providing little or no information about why and how an intervention worked (or not) and the context in which it was delivered [9]. Generalisability of findings may be limited, and interventions may be rejected if found to be ineffective or without a robust evaluation. Furthermore, knowing which components of an intervention are core, which components can be modified, and how they are delivered to produce an effect in a certain population is crucial for the resulting intervention to be adapted or scaled-up [11].

Multiple guides and tools are available to integrate implementation science concepts and methods to facilitate the identification and evaluation of factors likely to affect implementation efforts and evaluate implementation outcomes alongside the clinical impact [9,12,13]. Integrating mixed methods is crucial to understand why and how an intervention may work, how well it is delivered, for whom and in which circumstances. Effectiveness-implementation hybrid type 2 trials take a dual focus on assessing the effectiveness of an intervention as well as evaluating implementation efforts [14] and require a multidisciplinary approach [12]. These designs are increasingly recognised in the evaluation of health interventions and have the potential to speed up the translation of research findings into routine practice, ultimately enhancing public health impact [14], however their application in maternal and newborn health is very limited [15].

The **P**ilot study **O**f midwifery **P**ractice in **P**reterm birth **I**ncluding women’s **E**xperiences (POPPIE) study is a hybrid type 2 pilot RCT of a model of midwifery continuity of care linked with specialist obstetric care for women at increased risk of PTB in London (United Kingdom), and was accompanied by a nested mixed-methods evaluation which was informed by the Medical Research Council (MRC) guideline for complex interventions [9]. The aim of POPPIE was to determine whether this care model was feasible and could improve a composite outcome of timely and appropriate interventions provided for the prevention and/or management of preterm labour and birth. These included: antibiotics for suspected/confirmed urinary tract infections, transvaginal scan assessments of the cervix, fetal fibronectin assessments, cerclage insertion, progesterone administration, corticosteroid administration, magnesium sulphate administration, admission for observation, in-utero transfer, and smoking cessation and domestic violence referrals.

**Fig 1:**
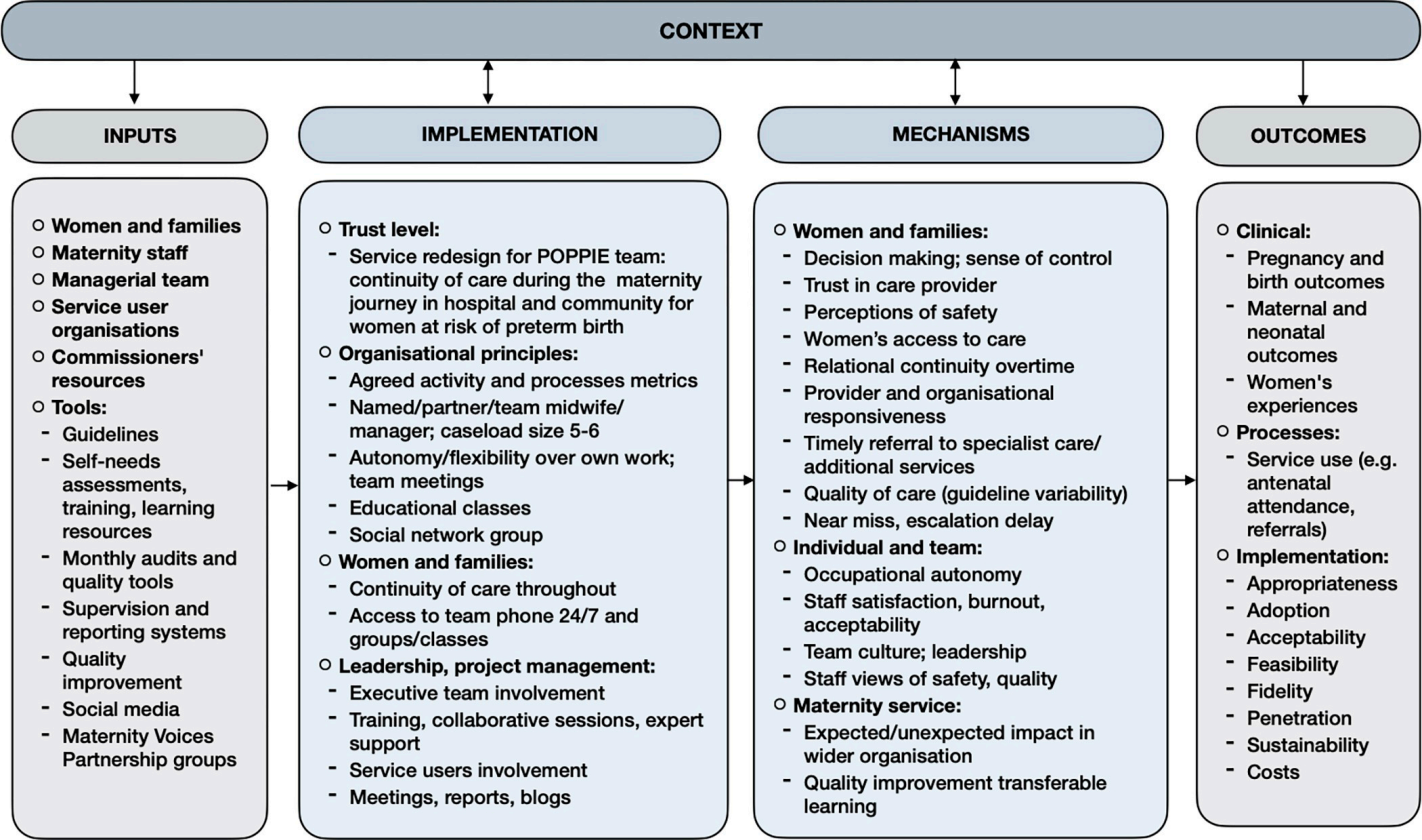


It was hypothesized that initiation of these treatments would occur earlier and at a more appropriate time in the intervention group, as the POPPIE model would improve quality of care by providing a trusted safety net with midwives ensuring better care coordination, referral, and access to additional services [16] and by moderating the effects of women’s stress on her health through trust and confidence that relational continuity generates [17]. Together, this would result in more women receiving timely and safer care leading to more opportunities for early identification of complications and subsequent management, thus improving prevention, detection and management of preterm labour and birth. [13,18]. A logic model was created in collaboration with local stakeholders, healthcare providers (HCP) and service users (Fig 1) to present these assumptions, processes, and anticipated outcomes, and inform key areas for evaluation in this study.

We report the evaluation of the implementation of the POPPIE model, describe the local context in which it was delivered, the mechanisms of impact by which the delivered model may produce change, and integrate results to determine whether the effect of POPPIE on the primary outcome in our setting could be explained. Specific objectives included:

- Implementation outcomes: to evaluate the perceived fit of POPPIE to address local needs (appropriateness) and intention to try its use (adoption); to determine whether POPPIE could be successfully carried out within this clinical setting (feasibility); to evaluate satisfaction among women, healthcare professionals and other key stakeholders with various aspects of the intervention (acceptability); to evaluate whether the intervention was implemented as outlined in the protocol and/or whether adaptations were made (fidelity); and to evaluate how well the intervention was integrated within the maternity system (penetration) and the extent to which it was maintained over time (sustainability)
- Context: to describe contextual factors which impact upon’ implementation efforts.
- Mechanisms: to explore if and how the intervention impacted on maternity care in this setting and identify possible reasons for this.
- Inter-relationships: to explore relationships between core implementation and process outcomes and the primary outcome.

## Methods

### Study design and participants

We used a multiphase mixed method triangulation pragmatic design nested within a hybrid randomised, controlled pilot trial [14,19]. This approach was specifically chosen to combine sequential and concurrent collection of multiple qualitative and quantitative data over four phases that together address the multiple purposes of the POPPIE model: 1) review of meeting records and key documents (quantitative and qualitative), 2) postnatal surveys with women (quantitative), 3) interviews with women (qualitative) and 4) interviews with HCPs and stakeholders (qualitative). Phases 1, 2 and 3 followed an almost sequential design, but phases 3 and 4 were partially concurrent [19,20].

We registered the trial with the ISRCT register (ISRCTN37733900) and used The CONsolidated Standards of Reporting Pilot Trials (CONSORT) (S1 Checklist) the Standards for Reporting Implementation Studies (StaRI) (S2 Checklist) and the GRAMMS reporting guidelines for mixed methods research [21]. The protocol and primary findings are published elsewhere [22,23], but in brief, this study was a two-arm hybrid type 2, randomised, controlled, unblinded pilot trial to compare midwifery continuity of care (POPPIE) with standard maternity care for women identified at increased risk of PTB. Pregnant women attending for antenatal care at less than 24 weeks’ gestation and residing in the hospital catchment area were eligible for the trial if they fulfilled one or more of the following criteria: previous cervical surgery, cerclage, premature rupture of membranes and/or preterm birth, late miscarriage; previous short cervix or short cervix this pregnancy, uterine abnormality, and/or current smoker of tobacco. Women aged less than 18 years at recruitment and those who had a multiple pregnancy or were already receiving care from a specialist midwifery team (e.g., severe mental illness, substance misuse) were excluded. Most pregnant women were recruited to the study by research assistants and midwives at their antenatal or ultrasound scan appointment. Others were referred by other HCPs or recruited from direct advertising at the hospital.

### Randomisation and masking

Women were randomly assigned in a 1:1 ratio via a secure web-based system (MedSciNet) to the intervention (POPPIE group) or control arm (standard group). A minimisation algorithm was used to ensure balance between the groups regarding previous PTB and smoking at booking. Blinding of participants and HCPs could not be achieved due to the nature of the intervention. However, study allocation was blinded to the statistician and the researchers who analysed the data [22,23].

### Interventions

The intervention, a caseload midwifery continuity of care model, is described in detail in the study protocol [22] but in summary, women allocated to the POPPIE group received antenatal, intrapartum and postnatal care in the hospital, community or at home, predominantly from a named (or primary) midwife, who worked with a partner midwife within a small team, known as the POPPIE team, under the supervision of a midwifery team leader. The POPPIE team was hospital based and had rapid access to a senior consultant obstetrician with expertise in PTB, who was allocated to the team to enhance clinical consultation and referral processes. Women allocated to the standard group received standard maternity care provided by different midwives working in the community, children’s centres and/or hospital. In accordance with the hospital guidelines, women in both POPPIE and standard groups followed the same obstetric care pathway [22].

### Follow up

Women in the POPPIE and standard groups who had a live birth and did not withdraw from the trial were followed up at six-eight weeks postpartum (or until discharge from neonatal intensive care unit up to three months). They were invited to complete a postnatal survey and participate in interviews based on a maximum variation sampling strategy [24] considering age, socio-demographic characteristics and obstetric history.

HCPs (frontline staff such as midwives, doctors) and other key stakeholders (i.e. senior management from the hospital, the local Authority, the Clinical Commissioning Group, Maternity Voices Partnership) involved in the development and/or delivery of the intervention were also invited to take part in a qualitative interview during mid and post-implementation. Both HCPs and stakeholders were recruited by non-clinical members of the research team using a snowballing approach through researchers’ and collaborators’ contacts. They were purposively selected to ensure participants were from different maternity settings, professional roles, and seniority levels.

### Ethics

Regulatory and ethical approvals were obtained (REC Ref 17/LO/0029; ID 214196), and written consent was provided by those participating in study.

### Outcomes

The primary clinical outcome of the pilot trial was a composite of timely interventions for the prevention and/or management of preterm labour and birth. Implementation findings were evaluated exploring key implementation outcomes, determinants of implementation and potential mechanisms of action, and were mainly informed by two major implementation frameworks: Proctor’s Conceptual Framework for Implementation Outcomes [25] and the Consolidated Framework of Implementation Research (CFIR) [26]. Given the complexity of maternity care models, CFIR was a very comprehensive framework to assess barriers and facilitators across multiple levels potentially influencing the implementation of a new continuity of care pathway in a hospital setting. Proctor’s was chosen as it is the only framework that allows a robust evaluation of the implementation of an intervention, and this was crucial to understand what processes and measures could be important to consider in a larger trial. An assessment of the potential impact of core implementation outcomes on the trial composite outcome is also provided.

### Data collection

**Fig 2:**
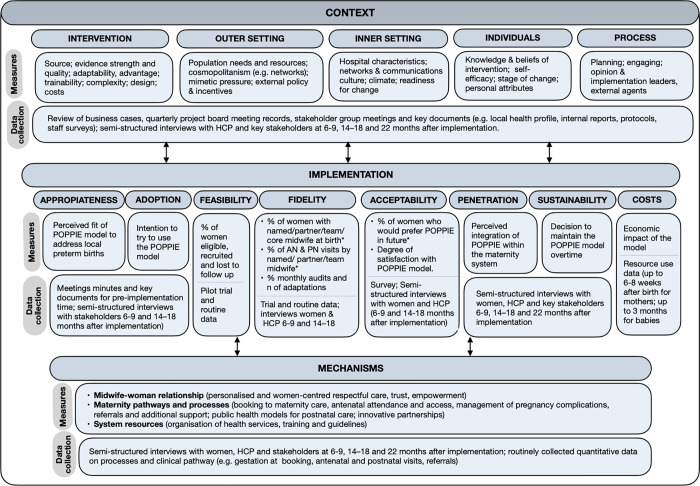


Overall, clinical and process outcome data were abstracted from medical records and electronic data systems, and implementation data (e.g. determinants of implementation, implementation outcomes and mechanisms) were collected from meeting records and key documents pre and mid implementation; and from postnatal surveys and qualitative interviews at 6–9, 14–18 months and post-implementation (22 months after implementation). Data collection for each outcome and determinants including measurement operationalisation are illustrated in Fig 2, and further detailed in S1 Table. The estimation of costs and resources are important outcomes of the implementation evaluation, but the economic evaluation for POPPIE will be reported separately.

Data from two business cases and records were collected from key documents (both public and provided by the implementation site e.g. local health profile, internal reports, protocols) and twelve executive and project board meetings (dates, duration, attendees’ roles, milestones and deliverables, timeframe, budget, risks) with directors, clinical managers, commissioners, public health specialists, academics and service users held over more than two years pre-implementation. Additional data were collected from more than fourteen monthly group meetings mid- and post-implementation with clinicians, researchers, implementers and users’ representatives (e.g. date, duration, attendees’ roles, project plan, workforce and funding arrangements, training needs, commitments, patient and public involvement, action points).

Postnatal surveys were completed by women at six-eight weeks after birth either online (through MedScinet) or by post (and posteriorly incorporated into MedScinet by research assistants). Up to three reminder phone calls and/or texts were sent every two weeks to non-responders. The survey, piloted with an advisory group (which included parents of preterm babies), was partially based on questionnaires from previous studies of models of maternity care and surveys conducted in the UK and included standardised scales to measure different aspects of women’s experiences of maternity care [27,28].

Semi-structured interviews were used as this style allows for a key list of questions to be addressed whilst providing flexibility for the interviewer to follow-up on points made by participants which are pertinent to their experiences [29]. Women (n = 30) were asked to share their experiences in their maternity care journey; and HCPs and local stakeholders (n = 23) were asked about their experiences in designing, implementing and/or providing the new care pathway. Interviews were conducted based on participants’ preferences (few over the phone and most face to face at home [for women] and hospital [for HCP/Stakeholders]) by three researchers with training and/or experience in qualitative methods not colleagues of participants and external to the hospital service provision. Reflective diaries were completed after each interview to encourage being continually reflexive [30]. Interviews lasted one hour on average and were digitally recorded and transcribed verbatim.

### Quantitative and qualitative analysis

Quantitative data was analysed in Stata version 15. For the primary outcome, the main analysis used binomial regression with a log link function to calculate the relative risks (with 95% confidence intervals). Adjustments were made for important factors associated with preterm birth (ethnicity, parity, education, index of multiple deprivation and obstetric risk). Details of randomisation and further analysis of the pilot trial are published in protocol [22] and primary results paper [23].

In order to evaluate whether there was a relationship between core implementation outcomes and determine whether this was related to the primary outcome, we used a ranking approach similar to previous public health studies [31,32]. First, quantitative implementation measures available for fidelity and acceptability (marked by an asterisk in Fig 2) were converted to a possible range (0 to 1) to give a score for each measure analysed. For example, in relation to ‘the proportion of women with the named / partner midwife attending birth’, a score of 1 was given if more than 50% of women gave birth with their named/partner midwife but a score of 0 was given if less than 50% of women gave birth with their named/partner midwife. Ranking scores we pre-defined based on some evidence available for fidelity (i.e. proportion of women receiving continuity models who were attended by birth by a known midwife varied between 63% to 98% in a Cochrane review [7]; thus pragmatic cut offs of at least 75% for any team midwife care, at least 50% for named/partner midwife care were used) (ranking approach in S2 Table). Then measures under the same domain were averaged and converted to a possible range (0 to 1) to give a score for each domain analysed (fidelity and acceptability). These were then averaged to give a single composite score reflecting their implementation strength (possible range 0–1) (averages used are arithmetic means throughout). Binomial regression with a log link function was also used to explore if the primary outcome was related to the individual and composite implementation scores.

Qualitative and quantitative data from meeting records and key documents were exported into excel and examined narratively. Qualitative data from interviews were uploaded and analysed in QSR NVivo version 12. The same interview data were analysed in three different datasets using three thematic frameworks appropriate for cross-disciplinary health research [33]: 1) Proctor’s Implementation Outcomes [25] were used as the thematic framework to analyse implementation outcome data; 2) CFIR [26] was used to explore the context; and 3) an overarching analytical framework was devised to explore potential mechanisms by using programme theories from a realist review of continuity of care and preterm birth (midwife-woman relationship, maternity pathways and processes, system resources) [34]. Coding followed each of the framework matrices, and once complete, data were stratified by participant type (women, HCP, stakeholders) to allow for differential interpretation, whereby the most relevant quotations were then reported in the text. To ensure rigour or ‘trustworthiness’ of the data, not only reflective diaries were used but transcripts were coded by two researchers and discussions and credibility checks were conducted for inconsistencies between the findings, the raw data and interpretation. The overall agreement was deemed to be high [35].

### Mixed-methods triangulation

A parallel approach was employed for data triangulation [36], whereby quantitative and qualitative data collection and analysis were undertaken separately for each of the four phases and brought together at the results and interpretation stages [37]. This pragmatic approach allows for quantitative and qualitative data to be integrated in a meaningful and complementary way that extends and clarifies each set of data increasing the value and function of the findings [38]. Analysis and interpretation of these integrated data was therefore exploratory, reflecting guidance for mixed methods pilot trials [39].

## Results

Between May 2017 and September 2018, 334 women were recruited to the main pilot trial; 169 women were allocated to the POPPIE group and 165 to the standard group. Of the 149 women followed-up in the POPPIE group, 90 (60.4%) completed the postnatal survey; and out of the 154 women followed-up in the standard group, 78 (49.4%) completed the postnatal survey. The overall survey response rate in both groups was 55%. A total of 20 women in the POPPIE group, 29 women in the standard group and 28 HCPs and key stakeholders were invited for an interview; and 16, 14 and 23 accepted (acceptance rate of 80%, 48.3% and 82.1% respectively). The overall interview acceptance rate was 70.1%. An overview of the characteristics of the qualitative participants are shown in Table 1.

**Table 1:** Characteristics of interview participants.

Timeline after implementation	6–12 months	16–20 months	24 months
**Women in the POPPIE group & Women in the standard group, n (%)**			
Maternal age group (years)	N = 16 & N = 14	N = 16 & N = 14	N = 16 & N = 14
<20	0 & 0	1 (6.2) & 0	0 & 0
21–29	1 (6.2) & 0	2 (12.5) & 1 (7.1)	0 & 0
30–39	3 (18.8) & 2 (14.3)	8 (50.0) & 8 (57.1)	0 & 0
>40	0 & 0	1 (6.2) & 3 (21.4)	0 & 0
Ethnicity			
White	3 (18.8) & 2 (14.3)	10 (62.5) & 9 (64.3)	0 & 0
Black	0 & 0	2 (12.5) & 3 (21.4)	0 & 0
Asian	1 (6.2) & 0	0 & 0	0 & 0
Mixed or other	0 & 0	0 & 0	0 & 0
Deprivation Index quintiles 1–2 (most deprived 40% of population)	2 (12.5) &1 (7.1)	2 (12.5) & 3 (21.4)	0 & 0
Highest educational level			
GCE or equivalent	1 (6.2) & 0	0 & 2 (14.3)	0 & 0
Vocational qualification	0 (0.0) & 0	2 (12.5) & 0	0 & 0
A level (or equivalent)	0 (0.0) & 0	1 (6.2) & 2 (14.3)	0 & 0
First degree	2 (12.5) & 1 (7.1)	5 (31.2) & 4 (28.6)	0 & 0
Higher degree	1 (6.5) & 1 (7.1)	4 (25.0) & 4 (28.6)	0 & 0
Pre-existing medical conditions			
Yes	2 (12.5) & 1 (7.1)	3 (18.7) & 3 (21.4)	0 & 0
No	2 (12.5) & 1 (7.1)	8 (50.0) & 9 (64.3)	0 & 0
**Healthcare providers and key stakeholders (N = 23), n (%)**			
Healthcare providers	2 (8.7)	9 (39.1)	0
Midwives	2 (8.7)	8 (34.7)	0
Doctors	0	1 (4.3)	0
Key stakeholder	2 (8.7)	10 (43.5)	1 (4.3)
Hospital	1 (4.3)	5 (21.8)	1 (4.3)
Clinical Commissioning Group	1 (4.3)	2 (8.7)	0
Local authority	0	2 (8.7)	0
Service use	0	1 (4.3)	0

Abbreviations: POPPIE, Pilot study Of midwifery Practice in Preterm birth Including women’s Experiences; GCE, General Certificate of Education.

### Implementation outcomes

#### Appropriateness

Most local stakeholders recognised PTB as a leading cause of child mortality in the area. A pre-implementation audit conducted by public health specialists confirmed a high number of early births and identified local complex needs. Consequently, the majority of HCP and stakeholders perceived the introduction of a continuity of care model as a fundamental change with possible benefits on local maternity services and preterm births, and decision to provide funding for a midwife leader post to lead organisational change was made. The most illustrative quotes on appropriateness and the rest of implementation outcomes are presented in Table 2.

**Table 2:** Selected qualitative quotes to illustrate implementation outcomes.

Implementation outcome	Illustrative quote
Appropriateness	Stakeholder, local authority, 033: “…most babies died because of poor outcomes of pregnancy, and most poor outcomes of pregnancy which resulted in children’s deaths were around prematurity… So we began to think very carefully about what we might recommend in terms of reducing the levels of prematurity….” Stakeholder, local authority, 024: “I think POPPIE wasn’t the only possibility but that was the one that was available, so, it seemed like it was worth a punt”. Stakeholder, clinical commissioning group, 025: “…we managed to cobble together some money to support, obviously largely supported by CLAHRC… I think £50k to support some of the, oh, I think the lead midwife, er I can’t remember exactly what it’s covering, but the midwife. Um, and so we sort of agreed that we didn’t go any, through any massive decision tree as such. It was more that, sort of a good thing to do. It linked with the STP [Sustainability Transformation Partnership] work we were doing anyway…” Stakeholder, hospital, 022: “We were really excited to be approached and at that point there wasn’t very much midwifery research going on in the Trust. There was quite a lot of obstetric, but not so much midwifery, so it was good to get a midwifery focused research. And, um, the senior midwives were also keen, because there was so much around offering continuity, and, and how to do it. But, huge concerns about whether we could do it. Because in that, in the Trust there hadn’t been any continuity models”
Adoption	Stakeholder, clinical commissioning group, 026: “I must admit, it was um, I mean I might be straying outside of the question, but it was such an uphill battle initially, getting it started at [Trust]. They’d not really had anything on that scale before.…” Stakeholder, local authority, 024: “But I think it was a really good learning, because it was an example of how, if you have lots of different people who, who all want it to happen, you can, with the levers, it’s not one particular thing that eventually makes it happen, it’s all these different things everywhere”. Stakeholder, hospital, 031: “It was quite difficult to begin with, and the, it was all being set up as to how it could happen, and we had to sort of release some of your whole-time equivalents from your teams to make the new team, and, and then to have, you know, women, so setting up the whole system here, it was, it was difficult to begin with, but, very soon it just became the norm”.
Fidelity	Woman 175: “I think, whenever, whether it was [midwife] or [midwife] who was here you, you know, I always sensed that I was their focus, their minds weren’t elsewhere on the next appointment or anything else… You know, following on from the last appointment they always knew what we’d discussed, whether there was a check-up I’d been to and anything to chat about. And there was definitely a trust relationship that developed through that continuity, that same person”. Woman 039: “I had [midwife] and I kind of just felt like it wasn’t just a health professional, I was with somebody who cared for me, basically” “And when it was needed, when they needed to act, they always acted very quickly, when it was important. . .” Midwife 013: “When we were very busy, often we only managed one on-call a night, where we always plan to have two on-call… but we always found there was usually somebody who offered to be the second midwife if we needed it. So, um, we work a lot on goodwill, especially when we’re short-staffed, or really busy…Um, but, at the moment we’ve often got some nights with only one on-call, and we’ve started now to use the community midwives as a second on-call if we need to at births. And, and that’s for home births, and that’s partly so that they don’t become deskilled, and also just to take the pressure off the team a little bit”.
Acceptability	Woman 123: “I think, you know, it was excellent, that’s the one word that comes to mind when I think back to my experience with them [POPPIE team], it was … you know, they went the extra mile in terms of care and support and it was, you know, it was really, really positive”. Midwife 010: “I think I have a lot of autonomy. And I have a lot of control over how I work. So in terms of my day to day job, and actually in terms of my own accountability for my hours and time, I don’t have someone who says to me, you were not here for this time, you know, or who wants to check my diary. Um, I think that level of trust is really important as well”. Midwife 005: “There was very much a culture, which is admirable, of, ‘I’ll come in on the weekend, even though I’m not on-call, because I’ll come and support you’, which is lovely, but I felt like, because the people higher up in the team had that way of working for themselves, that it was expected lower down, and I felt that like there weren’t enough boundaries… I actually wanted to be able to go home and close the door on work. And, I felt like I couldn’t do that, because you might get called at any point, or called, saying, there’s this lady just down the road, could you just see her? And I I didn’t want that level of intrusion. Um, and I also felt that, because of the model, the women had quite high expectations of your availability…”.
Penetration	Midwife 007: “I think one of the issues that I’ve perceived is that, as pre-existing community teams, we all know each other and we all kind of mesh and integrate, and we all share, our learning and stuff like that. But, I think the experience with, with POPPIE has been, because they’re caseloading, they don’t tend, and they’ve got their own space upstairs, there has been less sort of intermingling between the team members. And so there, I’m not even saying that there’s a them and us mentality, but I think there’s, it’s just lack of understanding”. Midwife 003: “Mm. . .the idea of being as independent and as autonomous an advocate, on the delivery suite, that, that sounds like that’s just not possible… but really hard…”. Woman 013: “It was almost as if you said that you were with the POPPIE team…just wait for them. We’re not going to do anything until that point… so there’s definitely some sort of disconnect there, which is a shame because everything else was so amazing.” Stakeholder, hospital, 023: “I think at the very beginning there were times where women were maybe coming in without calling the team themselves. And then the wards weren’t necessarily calling the POPPIE midwives. And I think that just needed to become embedded. And, I think that was probably the hardest bit… I think once everybody understood what the POPPIE team were doing, and that they were caseloading women, and, and how happy the women were, and wanted their POPPIE midwife with them, um, that worked really well. So that the wards, you know, the birth centre, or the labour ward, would call the POPPIE midwives in, or the women would let them know”. Stakeholder, hospital, 031: “I think the experience for the women, you know you can’t put a price on that. That’s the hard thing, how do you cost somebody’s experience? And I think that never gets taken into account…” “… and, you know, when we look at complaints, we don’t get complaints about the POPPIE team. You know, and when women have seen different midwives, they’re, you know, they’re not happy with this bit, that bit, nobody, you know, listened to me. You don’t get that, because they’ve got their advocate there”.
Sustainability	Stakeholder, hospital, 013: “Well we’ve taken on a new caseload now… with a new mixed risk criteria devised after discussion between managers about who would benefit… so, it’s um, women who are planning a home birth. Women with mild to moderate mental health, who don’t fit the criteria for the mental health team… women with disabilities or learning difficulties, women who’ve had a stillbirth, or a neonatal death, including cot death, umm.. previous preterm birth, but 34 weeks or less, whilst in the trial it was anything under 37. . .” “We want to maximise the number of women who can benefit, so, it was felt that women who’ve had a very preterm birth would get more out of it, because those are the babies that go to NICU for extended stays, and, it’s really helpful having the postnatal care from us, so that we can support them in NICU, and afterwards”. Midwife 008: “Being able to take who we want to take, really, we’ve got a lot more freedom. We’re not restrained by the trial, by the criteria of the trial…” “Also, we don’t have the added pressure of, of the trial, you know, getting the results of the trial, getting positive results, and also, you know, being, having to be there, or feeling like we absolutely have to be there”.

#### Adoption

There was a clear intention by most local stakeholders and HCPs to try to implement the model. However, some stakeholders did acknowledge numerous and tedious steps required for adopting the intervention in clinical practice due to lack of other continuity models and maternity service reconfiguration. The midwife leader was the only post externally funded and, initially, there were particular concerns on creating a new team with no additional staff (but a relocation of an already limited workforce or hospital funding for core staff recruitment) and lack of awareness of how a continuity team functions within the maternity care system of the hospital.

#### Feasibility

A total 553 women were screened for trial participation: 334 women met all inclusion criteria and were randomised (169 POPPIE group; 165 standard group), 123 did not meet the inclusion criteria and 96 declined to participate [23]. Eleven (6.5%) women in POPPIE and eight (4.8%) in standard group either moved out of area antenatally or postnatally or experienced in-utero transfers in threatened preterm labour to a tertiary hospital. Data for analysis was collected from 168 women (one woman withdrew, with consent to use all data withdrawn) in the POPPIE group, and 163 women in the standard group (two women lost to follow up) [23].

#### Fidelity

The named or partner midwife provided nearly 75% of antenatal and postnatal visits for women in the POPPIE group and was present at birth for nearly 57% of those women. A POPPIE midwife provided more than 85% of antenatal and postnatal visits and was present at birth for more than 80% of women in the POPPIE group [23]. Nearly 90% of POPPIE midwives submitted monthly audits to monitor own clinical activity and quality of continuity. Qualitative interview data on fidelity found that the majority of women in the POPPIE group met at least two to three midwives (and more than a third met most team members), they had a known midwife at birth, and a team member was available most times during pregnancy, birth, and the postnatal period. Qualitative interview data from HCPs showed that certain aspects of the intervention were adapted and tailored to meet certain needs in certain circumstances such as the organization of team’s on-calls from two to one per week during busy or under-staff periods, or the recommendation of further training on bereavement care.

#### Acceptability

Nearly 97% of women who completed the postnatal survey in the intervention group reported they would prefer a POPPIE midwife to be the main person for their maternity care. All women interviewed described their satisfaction with various aspects of the continuity model such as having direct access to their midwife and the team (mobile number to call and text anytime; flexibility in timing and location of visits); building a relationship over time (personalised advice, involvement of the family) and receiving coordinated care (consistency of information, practical support). Most midwives delivering the intervention highlighted positive elements of working in the POPPIE team including autonomy over own diary; job satisfaction through providing continuity and being part of research; a supportive team, and team culture, and access to training and development. Some reported flexible working and weekly on calls enhanced their work-life balance, whilst others reported exactly the same features disrupted their work-life balance, particularly during periods of sickness or annual leave, when the flexibility that the team depended on causes midwives to ‘stand in’ for each other on days off and to volunteer for ‘informal’ or ‘silent’ on calls. Overall, two midwives left the team but new team members were promptly recruited to replace them.

#### Penetration

When assessing the integration of the POPPIE model within the maternity services, many HCPs working on the new team encountered issues at the ‘boundaries’ between themselves and established services (especially labour ward and community teams) and potentially due to lack of knowledge about how the team worked (e.g. philosophy of care, visits, inductions of labour, on-calls). A couple of women also perceived a disconnection between their midwives and core hospital staff; they described care as being disjointed and observed unpleasant behaviours by core hospital staff towards POPPIE midwives.

#### Sustainability

Despite initial challenges in penetration, long term support for the continuity model remained high, amongst women, HCPs and stakeholders. Clinical managers recognised the importance of the support received by the POPPIE midwives when needed, the improved experiences of care among women looking after them and lack of complains from the team. A follow up interview with one of the stakeholders reported that the continuity model was sustained post-implementation and adapted to provide care to a mixed risk caseload of women, and felt current maternal policy on continuity of care facilitated the scale up of four continuity models targeting other local populations (e.g. vulnerable or socially disadvantaged women).

### Relationship between core implementation outcomes and the primary outcome

The effect of the intervention on the primary composite outcome was similar in the POPPIE group and standard group (RR 0.98; 0.90 to 1.08). After planned adjustments for important measurements associated with PTB (ethnicity, parity, education, deprivation, obstetric risk), no significant benefit of the intervention was found. None of the high- or low- fidelity and acceptability implementation measures were associated to the primary outcome. When measures were aggregated into a composite score, the combination of high fidelity and acceptability and low fidelity and acceptability were not significantly associated with the primary outcome (RR 1.21; 0.73 to 2.01 and RR 0.82; 0.49 to 1.35 respectively). The relationship between implementation measures and the primary outcome is detailed in S3 Table.

### Context of implementation

#### Intervention characteristics

Data from the initial business case and project meetings proved the intervention initially entered into the Trust through external sources (university and local public health department). However, the POPPIE model was then designed in partnership with researchers, clinical managerial teams, maternity commissioners, public health specialists and service users, after discussing at various meetings the strong evidence, quality, and cost-effectiveness behind continuity models. To avoid centrally driven and controlled leadership, researchers built a coalition between stakeholders through information sharing and decision making, conducted local consensus discussions to develop and agree on the characteristics of the POPPIE model (e.g. team size, targeted population), and also involved executive boards in the intervention development. A patient and public involvement group was actively involved in discussions of the intervention and outcome measures important to women at risk of PTB and their partners through biannual meetings, social media groups, and service user events. A relative flexibility of the POPPIE model was agreed but guarantee of core components (e.g. relationship continuity, autonomy over own work) was important. Thus, adaptability was promoted by allowing certain aspects of the intervention to be adapted and tailored (e.g. organisation of team’s on-calls, rota and workload; skills mixed; further training). A six-month piloting phase, led by the team leader, enabled researchers and clinicians to build experience and expertise in the complex reconfiguration of local maternity services before commenting the trial (see Table 3 for quotes to illustrate context of implementation).

**Table 3:** Selected quotes to illustrate determinants of context.

Determinants of context	
Domain	Qualitative quote
Intervention characteristics	Stakeholder 033, local authority: “… we decided we needed to do something about the prematurity… So, we, um, we began to look at the literature, to see what might be done.. But then, in the midst of all ponderings as to what we could do, we were approached by [colleague], I think, whether we would like to collaborate in a project which would be looking at the continuity of care and its impact on pregnancy outcomes, and in particular, prematurity. So that’s kind of where it came from…” Stakeholder 011, hospital: “But from originally being approached, I was really excited about it, because, continuity is, was, but I think makes a real difference to women… And, um, I think, you know, [colleague] um and [colleague], all of us, believe in caseloading, and we know that, historically, outcomes for women have been really good with caseloading. . .” Stakeholder 035, hospital: “And because we pilot the model for a bit before starting the trial, it did help out to actually, you know, get the team, let the, the hospital staff know about the team. Um. Yeah, but I don’t know if there was something as specific that actually, you know, I think it was again a combination of things, and er, yeah, and going to the meetings, to the band 7 meetings as well…” Stakeholder, Service user 021: “Every single woman, regardless of medical condition, every woman, should be entitled to continuity of care. Because, if it proves to be positive, then why shouldn’t it be the outcome for all?” “I assume it’s a funding thing as to why we can’t just, all women have it, and staffing levels possibly, but, yeah my view is that all women should have that level of care”.
Outer setting	Stakeholder, local authority, 021: “I think when we had the first conversation with them [commissioners], we just used a financial argument. You know, the expensive cost of a baby in NICU [Neonatal Intensive Care Unit] and also, I mean, unfortunately and fortunately, we had some data that showed we had the second highest rate of preterm birth in [city]”. Stakeholder, clinical commissioning group, 026: “Everything just happened before the impetus about continuity of care from Better Births [policy]” “But I suppose it’s been helpful cause things have moved on in a sort of quite supportive direction for caseloading”. Stakeholder, hospital, 031: “We are looking at how we can expand the continuity model of caseloading… at different ways that we can do it. It definitely needs to expand. Partly because we know it’s really good for women, good for outcomes. But also, because that is what we’ve been directed from by the Better Births [policy]”
Inner setting	Midwife 003: “I was kind of worry about that a little bit, really, ‘cause I, I think there’s lots of evidence that shows that continuity of care promotes normality and improves normal birth rates. But I think that you need to look at it within i-, context, and if you’re working in an environment like here, it’s really hard… quite a high-risk setting” Midwife 007: “So I think, definitely in the beginning it was, it was a real challenge to spread the message of, of how the POPPIE team fitted in, as an addition to what was already being provided by the hospital”. Stakeholder, hospital, 031: “Well, we wanted to increase our research here at [Trust] anyway. So, that was the first thing, we wanted a couple of research projects. We very much believed in the caseloading model which we wanted, and POPPIE fitted both”
Participants involved	Stakeholder 022, hospital: “There weren’t really any midwives in the Trust that had even come from another Trust that had done it [continuity]. And although lots of midwives when we spoke to them said they were interested, it was always the how, you know, how are we gonna do it, and how much is it gonna cost? Were the big concerns. . . And, when we put adverts out and stuff, responses were good, but lots of people didn’t attend for interviews, or weren’t suitable, so we had a small number of midwives in the end that we could put into the trial”. Stakeholder 025, clinical commissioning group: “But clearly there are challenges, the whole workforce generally, and um, so that’s not from POPPIE, but from the whole, you know, midwifery issues… suppose it’s sort of how far POPPIE drains any midwives away from the core service. I’m not aware of any issues, but I suppose any reflections on that would be helpful to consider as part of the research or evaluation…. so it’s just that, that bit of the configuration, but if it helps to answer shift patterns, work patterns, which have been an issue, as to how we support midwives, as well as the mums, to, you know, burnout, support, risks and things.” Midwife 012: “I think we had recruitment problems initially, ‘cause it took us nearly a year to recruit to the team. But now, actually we had a couple of people that have relocated, and, those posts were filled relatively easily by internal midwives who were interested…”. Midwife 016, hospital: “This work was giving me, I think an extra chance of learning different environment, as the preterm birth support, and, um, other fields around midwifery and research”
Process of implementation	Midwife 013, hospital: “So, at the beginning, we started to caseload women who would fit the criteria for the trial, but without starting the research, just to try and get things going really. . . Just to sort of see how it could work, and to iron out problems and trying to negotiate what it would look like…”. Stakeholder 024, local authority: “Well I think it [the study] was very well run, from the [research organisation] point of view… there have been very very regular meetings, and all of that, and you know that’s probably standard, but it’s pretty crucial… They stuck with it”

#### Outer setting

The review of local documents revealed that approximately 30% of the local population are from Black and minority ethnic groups and levels of deprivation were higher than the national average. Health profile reports from Public Health England at pre-trial time found that there were more than 6000 births in the hospital and approximately 8.2% of women delivered preterm babies, which is slightly above the UK rate of 7.8% [40]. Important financial implications related to local PTBs and associated child mortality were acknowledged by most stakeholders. In addition, findings from the pre-implementation audit identified a care and quality gap and encouraged maternity commissioners to fund clinical innovation, developing a financial formula for the midwife leader post to implement the new care pathway. National maternity policy set up just before the pilot trial commenced and recommending scale up of high-quality continuity of care [41] was a strong facilitator for the implementation and sustainability of POPPIE, as well as the NHS Long Term Plan endeavouring continuity of care for most women [42], and the WHO antenatal and intrapartum guidelines for positive pregnancy experiences highlighting the benefits of continuity in settings with well-trained midwives [43,44]. National policy might have affected clinical practice in standard care group, particularly during the antenatal period when more than half of the women were seen by the same two midwives. The implementation of POPPIE was also influenced by a robust network between maternity services and other organisations (e.g. maternity networks, commissioning and strategic clinical groups, university). There was presence of mimetic pressure from competing organizations who already implemented similar care models and events were organized for management and leadership tips from external managers who had successfully reconfigured services at other hospitals.

#### Inner setting

Local documents reported that the hospital had quality improvement processes in place (e.g. sharing on maternity key performance indicators, staff survey results) and its vision and mission statement included three key principles underpinning the care delivered at every stage of their maternity pathway: informed choice, accessibility and continuity of care [45]. Although there were no existing continuity models in the hospital, there was an organisational commitment and a shared vision among hospital managers, commissioners, and service-users of the importance of implementing this care pathway. Thus, the organisation mandated a change by highlighting in the maternity services specification report their formal commitment to deliver high quality continuity of care for women. A piloting phase of 6–8 months, led by the midwife team leader, enabled stakeholders and care providers to build experience and expertise in reconfiguring local services before commenting the trial (e.g. in terms of workforce, pathway for referrals). To actively support adoption and raise awareness of sustainability of similar models, multiple implementation strategies were used e.g. sharing of experiences with other hospital managers; using advisory boards and workshops to monitor progress and review risks; and sharing educational materials (e.g. standard operating guidelines continuity of care toolkit, preterm birth prevention). Similarly, to address some of the initial penetration challenges and enhance positive relationships with managers and colleagues working in other services, other strategies were used e.g. attendance to clinical, community and multidisciplinary meetings; sharing of relevant team materials (e.g. operating guidelines); and support of core maternity services when possible.

Interviews with HCPs and analysis of local documents found a lack of research culture in the maternity services and the need for innovation was recognised. Thus, the organisation mandated a change by highlighting in the maternity services specification report their formal commitment to deliver high quality continuity of care for women and started organisational changes (e.g. layout change of several rooms to provide a small office space for the team, new equipment). The implementation of POPPIE meant the hospital became actively engaged in maternal health research and started to participate in clinical trials in collaboration with other sites. At the same time the trial was being set up, a preterm surveillance clinic was also being established as the main obstetric care pathway for women identified at risk of preterm birth and this could have improved clinical management and quality of care for the study population.

#### Characteristics of individuals involved

In stakeholder group discussions, continuity of care was not supported by all HCP and some midwives did not want to work in the context of continuity models with the belief that they would experience burnout (e.g. with on-call system), they would perceive difficulties in autonomous decision making (e.g. particularly if only worked in routine models) or they would be routinely pulled to cover conventional service needs (e.g. labour ward, community). Recruiting and retaining midwives in Trusts is often difficult and recruiting midwives to implement these care models can be a challenge [35]. Two job advertisements were required and promotion of a learning culture, development and enhancement of research and clinical skills though the continuity model was emphasised. To avoid potential inter-professional tensions, both stakeholders and the team proposed it would be beneficial to introduce medical and midwifery teams to work together on the implementation project, with the aim of promoting positive relationships amongst and between teams.

#### Process of implementation

Planning, developing, and piloting the care model in advance enabled local stakeholders to work together to build capacity and expertise and provided time to reflect and support each other’s learning before commencing the trial, thus promoting adoption of the POPPIE model. Weekly and monthly research and clinical meetings were scheduled to find practical solutions to possible implementation challenges for women, midwives, and healthcare service. For quality and monitoring purposes, daily/queries logs with agreed action plans were in place, and monthly audit tools were designed to monitor fidelity of the intervention. Throughout the project a stakeholder engagement strategy was utilised a combination of social marketing, training and education, and other similar activities and involvement of team leaders, champions, project coordinators and services users.

### Mechanisms of action

#### Midwife-woman relationship

Women in the POPPIE group reported how important it was for them to have a trusting relationship with a midwife who provided individualized and respectful care for them and their families; and how reassuring it was to have telephone access 24/7 to a team, with whom they were familiar, for personal advice and support. They felt at the centre of their care, involved in discussions and informed choices, with no need to repeat own story every time which made them feel safer and calmer, and appreciated the advocacy through their midwives e.g. during birth choices or interactions with other health professionals. The importance of relationships as a pathway for safe and quality of care was also recognized by midwives and stakeholders (quotes to illustrate mechanisms in Table 4).

**Table 4:** Selected quotes to illustrate mechanisms of action.

Theory	Qualitative quote
Woman-midwife relationship	Woman 123: “I always sensed that I was their focus, their minds weren’t elsewhere on the next appointment or anything else… You know, following on from the last appointment they always knew what we’d discussed, whether there was a check-up I’d been to and anything to chat about. And there was definitely a trust relationship that developed through that continuity”. Woman 033: “I feel, I felt very, very supported and very, very cared for the whole way through, like, there wasn’t, there wasn’t a moment where I felt like, um, oh, I wasn’t worried at any point, I felt so confident in the care that I was having. I, I felt very, very valued and very, very cared for the whole way through.” Woman 011: *“*.* *.* *.But as soon as [midwife] was there, I stopped feeling fear, and like your body obviously relaxes. I think that without the fear there it would just, you just feel a lot safer anyway. And, and I think having had that experience I, I would now trust my own instincts. . .” Stakeholder, hospital, 024: “Um, oh I, I think, undoubtedly it’s the best model of care for women who are vulnerable in any way, regardless of it being a research project… I’m surprised at how women actually manage to get out of the door sometimes, with all of that going on, so, if they’ve got a relationship with a midwife, it’s got to make a difference”. Stakeholder, service user 021: “…once you’ve built a relationship with someone where you trust them, you know, maybe the third, fourth or whatever appointment, you feel comfortable enough to share things. So that’s gonna alleviate a load of pressures, um, for that woman. You feel, that’s OK, this person, I can, I can ask them anything… They’re not gonna judge, they’re not gonna make me feel bad, so… having that face that you know, or voice at the end of the phone even, to just ask a quick question….”
Maternity pathways and processes	Woman 037: “Nobody seemed to be too rushed, there was plenty of time. The appointment times were really handy, so I was on a temporary contract at the time and not getting paid for time off, so the fact that we could have appointments after work or on the weekends made a huge difference, you know, to me”. Woman 175: “…You feel less scared because you’ve got that constant reassurance, and information, like they’re constantly, I could text [midwife] and say, you know, ‘Are my bloods back?’ and she’d text back and say, ‘Yeah all clear.’ And it’s like, great, I don’t have to wait for a doctor’s letter, I don’t have to, it’s that kind of constant information and then, yeah, the consistency of just having somebody”. Woman 197: “I would contact [midwife] occasionally by text if say she’d done a urine test and sent it off and would text me saying it was fine. Or just to check if, you know, I’d had a scan just to see how that was, I’d text and say they were all fine. I had a couple of growth scans so she would always follow up after that”. Woman 023: “… it was that support in first two weeks particularly, having someone who I knew who, you know, to be there to, even if it is just for a phone call or to be reassured, you know, I think just made such a difference. I think obviously kind of breastfeeding is a much, can, you know, have a much bigger longer effect”. Woman 197: “It even got to a point that [midwife] even have to fight with the doctor for them to change my medication because they didn’t want to change it, I was on labetalol, but she thought it wasn’t working on me, that Nifedipine is actually better… She helped me to come back on that day that she’ll have a favourite doctor there that she can speak to, and that one changed it, yeah. . .” Doctor 032: “We had more of informal chats about say for example, if they’re getting a patient on a day, and I would say OK, I’m on the ward, let’s meet her in DAU, or I’m on labour ward, let’s do this, so, you know, there’s a formal bit to it, but there was more of a good teamworking and informal chatting and kind of getting our work around it, rather than, you know, she has to be seen in the clinic, or we have to do it this way”. Midwife 007: “I did definitely feel hostility on labour ward. . . I found people to be quite unhelpful. It would always be a real struggle to get relieved for a break, actually it didn’t really happen at all, and to actually get relieved when the shift had ended as well, or when the sort of episode of care had ended. They weren’t very good at, at letting you go home”. Stakeholder, hospital, 031: “I think you’re always gonna get a few issues with a midwifery team and obstetricians. There’s always gonna be a little bit of battling, because, when you’re really advocating for a woman, sometimes you have to battle a little. So I think there was a little bit of that at times. I think until people got to know the team, and got to know the midwives, because a lot of the midwives were new to our Trust. So, they had quite a hard job really, because they were coming to a new Trust, a new trial, a new way of working, so I think they did have to show their worth, almost, I think, and I think it was quite hard at times. I don’t think it was so much with the midwives. I think, um, the midwives could see the benefit and the expertise from early on, but I think, obstetric-wise I know the girls have had a few battles, at times”.
System resources	Woman 171: “The resources are stretched so thin, and you know, it’s a real struggle I think for, for people to give you anything. I think, I think all the midwives you see in your pregnancy really want to give you the best care possible, but they’re just so over-worked.”

#### Processes and clinical pathways

Most women accessed maternity care relatively early (mean gestation at booking 10.2 weeks, SD 2.7). However, timing of booking to care is unlikely to be affected by the intervention as nearly all women were recruited after the first antenatal visit with a community midwife or scan appointment (mean gestation at randomisation 15.1 weeks, SD 3.5). Women felt supported with access to midwives at any time and more informal and flexible visits (e.g. more time, home visits, re-arrangements) and reported they could openly discuss more sensitive or personal circumstances (e.g. vaginal infections, mental illness). The majority of these women had medical and/or obstetric risk factors and reported experiences of management of complications (e.g. urine infections, induction of labour, pre-eclampsia); and highlighted how timely interventions and referrals to other professionals were done. Details of process outcomes (e.g. total number of visits, total inpatient nights, total number of referrals) are provided in S4 Table. Women in the POPPIE group were more likely to be referred to the safeguarding team–a multi-agency team that protects women and their unborn child’s health, wellbeing and human rights to avoid harm, abuse and neglect (main reasons being housing, social care, and mental health) although safeguarding referrals and overall antenatal referrals were not related with the primary outcome (RR 0.62, 0.36 to 1.06; and RR 1.25, 0.78 to 1.98 respectively). They were also more likely to have more postnatal visits, and women highlighted during the interviews the practical and emotional support received during the postnatal period (especially with breastfeeding). Some women and midwives reported feelings of frustration due to poor organization, communication and cooperative relationships between HCPs working in different wards or departments (e.g. hospital wards, community, neonatal units) particularly at time of labour and after birth.

#### System resources

Lack of sufficient and/or trained staff working in a stretched, overworked and underfunded NHS was acknowledged by few women and may have impacted on delays on receiving support and elective and emergency interventions (e.g. inductions, cesarean sections).

## Discussion

This study reports the results of a mixed methods evaluation alongside a hybrid type 2 pilot RCT. The introduction of a model of midwifery continuity of care linked with specialist obstetric care for women at increased risk of PTB (POPPIE) was found to be appropriate to address local needs in a UK inner-city maternity service despite challenges in early adoption (e.g. lack of similar models, service reconfiguration). Collaborative working and availability of additional funding certainly facilitated adoption efforts. We demonstrated it is feasible to set up and maintain appropriate levels of fidelity of the POPPIE model with overall good acceptability among women and HCPs in both our quantitative and qualitative analysis. The model was eventually embedded within established maternity services and, after the trial, it was sustained and adapted to provide care to a mixed risk caseload of women with further scale up of other continuity models. Process outcomes were also similar in both models of care. Overall, we found no association between specific implementation and process measures and the primary outcome.

Implementation fidelity, thought to be critical to successful translation of evidence-based interventions into practice [46], was prospectively identified as a key measure used to address and change implementation concerns during the trial to maximise the likelihood of potential scale-up of similar continuity models in other contexts. We retained core components of the intervention in terms of relational continuity (e.g. visits with named/partner/team midwife, midwife at birth) and demonstrated that it was possible to adapt certain components of the model (e.g. on-calls, training) whilst maintaining a very high proportion of the intervention fidelity and acceptability among women. Although support for the POPPIE model also remained high throughout implementation among midwives and clinical managers, work-life balance was sometimes hard to maintain with “silent” on calls for labour care and cover for each other on days off, particularly during busy periods, and this could have potentially influenced implementation fidelity. These findings are aligned with inconsistent evidence on the impact of flexible working in continuity models among midwives, some studies suggest it increases wellbeing and satisfaction [47,48] whereas others suggest it increases burnout [49,50]. It is possible that these models may work for some midwives but not others (e.g. with caring responsibilities) [51]. Although the extent of support midwives have (e.g. with childcare, specially overnight or during unsocial hours) and their individual ability to ‘relax’ or ‘switch off’ during down time is likely to play an important role.

Despite multiple strategies adopted, the embedment of the model within existing services required longer than expected, and collaboration and good communication among HCPs were crucial to address issues in relation to how the POPPIE team worked. Although this was not very surprising; until recently, there was very little guideline on implementation and scale-up of continuity models, and the introduction of a first continuity model in our context, involved a complex, large-scale transformation of the organisation of maternity services requiring whole system support and close alignment between top and frontline leadership [13]. Eventually, maternal policy on continuity of care strongly facilitated the penetration and sustainability of the POPPIE model with the scale up of additional continuity teams planned based on other local women’s needs.

This is one of the few studies in maternity care (and the first of a midwifery continuity of care model) that rigorously uses implementation frameworks and mixed methods research to examine multiple process measures and analyse them alongside the primary outcome [15]. We demonstrated that evaluation of implementation and process outcomes alongside a pilot trial is feasible and valuable in understanding quality and quantity of diverse aspects of the implementation and exploring potential mechanisms of impact in our specific setting. We provide invaluable learning on implementation strategies and scale-up of continuity models, crucial for NHS England plans to expand midwifery continuity of care pathways for the majority of women by 2025 [42].

Although high quality implementation has previously been associated with better outcomes on individual level in health promotion programmes, it may not be the case for organisational-level implementation measured in a multidisciplinary healthcare context [52]. Actually, aligned with larger studies of maternal and primary health interventions [31,53] we did not find any correlation between core implementation outcomes and the primary outcome. However, this must be interpreted with caution; this was a trial designed for feasibility and with limited power to detect significant improvements in clinical or processes outcomes. Therefore, it is possible there was limited power to detect a significant relationship between the primary outcome and implementation measures. Understanding variability in implementation of complex interventions is important and future research is needed to determine how useful existing or new ranking approaches are for measuring implementation strength.

Nearly a third of women in the pilot study were from ethnic minority groups, more than two thirds lived in areas of social deprivation and more than a quarter had at least one pre-existing medical condition and multiple obstetric risk factors for PTB [22]. Hypothesised mechanisms as to how the model might work (improved care coordination and referral with engagement and access) do appear to have limited influence where there are pathological physiological mechanisms influencing PTB. It is possible that relational continuity would be more likely to improve outcomes in other populations suffering social determinants of PTB such as socially disadvantaged communities (e.g women who find services hard to access). Some mechanisms of impact identified during the qualitative evaluation (e.g. access, safety, trust, advocacy) may be limited to a part of the study population group due to limited socio-demographic and social risk diversity among interview participants [54]. Although it is known that people from Black and minority ethnic and socially disadvantaged groups are less likely to engage in research follow ups [55]. Our findings are similar to a mixed-methods comparison of women’s experiences that found that POPPIE improved experiences of trust, safety and quality of care compared to standard care, but further research should focus on women with more complex needs who have multiple social risk factors [54].

It is also possible that community-based models of care that focus on local people and communities and enhance place-based level of health and social care services can have a different impact on clinical and processes outcomes and inequalities when compared to hospital-based care models like POPPIE [56,57]. Recent pioneering Australian research with Indigenous women has shown an effect on preterm birth of a collaborative model of midwifery continuity of care integrated with family services and a community-based hub [58]. Aligned with current maternal policy on midwifery continuity of care models to improve safety and quality of care for women living in diverse and socially disadvantage areas, further investigation is required to evaluate the role of community-based models among women with complex social factors and vulnerability and to understand their experiences of care, potential mechanisms, and the long term impact. To do so, supporting and increasing community participatory research of diverse and socially disadvantaged groups is a key initial step [59].

## Conclusion

To our knowledge, POPPIE is the first hybrid type 2 pilot trial of a model of midwifery continuity of care for women at risk of preterm birth London (UK) that has evaluated core implementation and processes outcomes, using a mixed-methods approach, and integrating these with the primary outcome in order to understand the findings in our context. We have demonstrated that, overall, the POPPIE model of care was appropriate, feasible and implemented with high fidelity; was acceptable among women and HCPs; and was embedded, adapted, and sustained in maternity services. As expected for a pilot study focused on feasibility of a larger trial and with limited statistical power, there were no differences in the primary outcome and there was no association between implementation outcomes and the primary outcome. Larger appropriately powered trials considering primary outcomes such as preterm birth are feasible and needed, including in community settings, to evaluate the impact and implementation of continuity models in other populations such as women with social disadvantage and vulnerability.

## Supporting information

S1 Checklist (DOC)

S2 Checklist (DOCX)

S1 Table Measures and data collection methods and time points. (DOCX)

S2 Table Implementation measures and scoring approach. (DOCX)

S3 Table Relationship between core implementation outcomes and the primary outcome. (DOCX)

S4 Table Additional process outcomes for mothers and babies. (DOCX)
