# Supplementary material for: A continuity of care programme for women at risk of preterm birth in the UK: Process evaluation of a hybrid randomised controlled pilot trial
Source: PLoS One. 2023 Jan 12;18(1):e0279695. doi: 10.1371/journal.pone.0279695 (PMC9836307; doi:10.1371/journal.pone.0279695)
Supplement: S1 Table — (DOCX) [file pone.0279695.s003.docx]

**S1 Table: Measures and data collection methods and time points**

| **Implementation outcomes, determinants domain and mechanisms of action** | **Operationalisation** | **Data collection methods** | **Data collection time points** |
| --- | --- | --- | --- |
| Appropriateness and adoption | - Perceived fit of POPPIE model to address local needs;  - Intention to try to use the model | - Data from two business cases and records from twelve executive and project board meetings with directors, clinical managers, commissioners, public health specialists, academics and service users.  - Qualitative interviews with key stakeholders | - Pre-implementation  - 6-12 months |
| Feasibility | - Eligibility rates  - Recruitment rates  - Attrition rates | - Pilot trial data and routinely collected data | - 24 months  (monthly checks) |
| Fidelity | - Proportion of antenatal visits provided by the named/partner midwife  - Proportion of antenatal visits provided by another team midwife  - Proportion of women with the named / partner midwife attending birth  - Proportion of women with a team midwife attending the birth  - Proportion of postnatal visits provided by the named/partner midwife  - Proportion of postnatal visits provided by another team midwife  - Proportion of completed monthly quality audits  - Total number of adaptations of the intervention | - Pilot trial data and routinely collected data  - Data from monthly quality audit tools to measure continuity  - Qualitative interviews with women and healthcare providers | - 6-12, 16-22 and 24 months  (monthly checks) |
| Acceptability | - Proportion of women who would prefer a POPPIE midwife to be the main person for their maternity care if they were to have another baby  - Degree of satisfaction with the model. | - Postnatal survey data  - Qualitative interview data from women and healthcare providers | - 6-12 and 16-22 months |
| Penetration and sustainability | - Perceived integration of POPPIE within the maternity system;  - Maintenance overtime | - Qualitative interviews with all (women, healthcare providers and key stakeholders) | - 6-12, 16-22 and 24 months |
| Contextual factors | - Intervention characteristics (e.g. source; evidence strength and quality; adaptability, advantage; trainability; complexity; design; costs)  - Outer setting: population needs and resources (e.g. socio-demographics, health/wellbeing outcomes); cosmopolitanism (e.g. networks with external organisations); mimetic pressure from similar organisation; external policy and incentives (e.g. national maternal policy)  - Inner setting: hospital characteristics (e.g. size, vision, organisation); networks and communications (e.g. leadership, meetings); culture (quality performance indicators; staff satisfaction); climate (e.g. need for innovation); readiness for change (e.g. commitment)  - Individuals involved (e.g. Knowledge & beliefs of intervention; self- efficacy; stage of change; personal attributes)  - Process of implementation (e.g. Planning; engaging; implementation/  opinion leaders, external agents) | - Data from more than fourteen implementation monthly group meetings with clinicians, researchers, implementers and users’ representatives  - Key documents (e.g. local health profile, internal reports, protocols, staff surveys)  - Qualitative interviews with healthcare providers and key stakeholders | - 6-12, 16-22 and 24 months  (monthly checks) |
| Mechanisms of actions | - Midwife-woman relationship (personalised and women-centred respectful care, trust, empowerment)  - Processes and clinical pathways (booking to maternity care, antenatal attendance and access, management of pregnancy complications, referrals  and additional support)  - Postnatal care pathways (public health models, integration of maternity and family services)  - Collaborations and partnerships (innovative partnerships, co-development, shared leadership, community-based hubs)  - System resources (organisation of health services, training and guidelines) | - Qualitative interviews with women, healthcare providers and stakeholders  - Routinely collected quantitative data on processes and clinical pathway (e.g. gestation at booking, antenatal and postnatal visits, referrals) | - 6-12, 16-22 and 24 months |
