# Supplementary material for: A continuity of care programme for women at risk of preterm birth in the UK: Process evaluation of a hybrid randomised controlled pilot trial
Source: PLoS One. 2023 Jan 12;18(1):e0279695. doi: 10.1371/journal.pone.0279695 (PMC9836307; doi:10.1371/journal.pone.0279695)
Supplement: S2 Table — (DOCX) [file pone.0279695.s004.docx]

**S2 Table: Implementation measures and scoring approach**

| **Implementation domain: Fidelity** | **Ranking score*** |
| --- | --- |
| Implementation measures: |  |
| Proportion of antenatal visits provided by the named/partner midwife | 1 if > 50%  0 if <50% |
| Proportion of antenatal visits provided by another team midwife | 1 if < 25%  0 if >25% |
| Proportion of women with the named / partner midwife attending birth | 1 if > 50%  0 if <50% |
| Proportion of women with a team midwife attending birth | 1 if < 25%  0 if >25% |
| Proportion of postnatal visits provided by the named/partner midwife | 1 if > 50%  0 if <50% |
| Proportion of postnatal visits provided by another team midwife | 1 if < 25%  0 if >25% |
| *Overall implementation fidelity* | 1 if > 0.75  0 if < 0.75 |
| **Implementation domain: Acceptability** |  |
| Proportion of women who would prefer a POPPIE midwife to be the main person for their maternity care if they were to have another baby | 1 if > 75%  0 if < 75% |
| *Overall implementation acceptability* | 1 if > 0.75  0 if < 0.75 |
| **Implementation Composite Score** | 1 if > 0.75  0 if < 0.75 |

*Ranking scores we pre-defined based on some evidence available for fidelity (e.g. proportion of women receiving continuity models who were attended by birth by a known midwife varied between 63% to 98% in a Cochrane review; thus pragmatic cut offs of at least 75% for any team midwife care, at least 50% for named/partner midwife care were used).
