# Supplementary material for: A continuity of care programme for women at risk of preterm birth in the UK: Process evaluation of a hybrid randomised controlled pilot trial
Source: PLoS One. 2023 Jan 12;18(1):e0279695. doi: 10.1371/journal.pone.0279695 (PMC9836307; doi:10.1371/journal.pone.0279695)
Supplement: S3 Table — (DOCX) [file pone.0279695.s005.docx]

**S3 Table: Relationship between core implementation outcomes and the primary outcome**

|  | **POPPIE care**  **(n=168)** | **Standard care**  **(n=163)** | **Risk ratio:**  **(95% CI)** |
| --- | --- | --- | --- |
| **Primary clinical outcome** |  |  |  |
| Composite (initiation and timing of 1 or more interventions for the prevention and/or management of possible preterm labour birth*) | 140/168 (83.3) | 138/163 (84.7) | 0.98 (0.90 to 1.08) |

Data are n (%). * Antibiotics for urinary tract infections, transvaginal scan assessments, fetal fibronectin assessments, cerclage, progesterone, corticosteroid, magnesium sulphate, admission for observation, in utero transfers, smoking and domestic violence referrals. Abbreviations: CI, confidence interval.

| **Relationship between high acceptability and fidelity implementation and the primary outcome** | | | | |
| --- | --- | --- | --- | --- |
|  | **Ranking score approach*** | **Ranking score=1**  **(n=168)** | **Primary outcome**  **(n=168)** | **Risk ratio (95% CI)** |
| **Implementation fidelity measures** |  |  |  |  |
| Proportion of antenatal visits by:  Named/partner midwife  Other team midwife | 1 if > 50%  1 if < 25% | 152 (90.5)  163 (97.0) | 97 (57.7)  105 (62.5) | 0.85 (0.62 to 1.15)  0.80 (0.51 to 1.26) |
| Proportion of births attended by:  Named/partner midwife  Other team midwife | 1 if > 50%  1 if < 25% | 94 (55.9)  42 (25.0) | 60 (36.7)  24 (14.3) | 0.96 (0.77 to 1.20)  0.84 (0.63 to 1.13) |
| Proportion of postnatal visits by:  Named/partner midwife  Other team midwife | 1 if > 50%  1 if < 25% | 136 (80.9)  98 (58.3) | 90 (53.6)  66 (39.3) | 1.11 (0.81 to 1.52)  1.09 (0.87 to 1.38) |
| *Overall implementation fidelity (all measures)* | 1 if > 0.75 | 120 (71.4) | 81 (48.2) | 1.12 (0.90 to 1.39) |
| **Implementation acceptability measures** |  |  |  |  |
| Proportion of women who would prefer a POPPIE midwife to be the main person for their maternal care if they were to have another baby | 1 if > 75% | 86/89 (96.7) | 54/89 (60.7) | 0.94 (0.4 to 2.13) |
| **Implementation Composite Score** | 1 if > 0.75 | 74/89 (83.1) | 48/89 (53.9) | 1.21 (0.73 to 2.01) |

| **Relationship between low acceptability and fidelity implementation and the primary outcome** | | | | |
| --- | --- | --- | --- | --- |
|  | **Ranking score approach*** | **Ranking score=0**  **(n=168)** | **Primary outcome**  **(n=168)** | **Risk ratio (95% CI)** |
| **Implementation fidelity measures** |  |  |  |  |
| Proportion of antenatal visits by:  Named/partner midwife  Other team midwife | 0 if < 50%  0 if > 25% | 16 (9.5)  5 (2.9) | 71 (42.3)  63 (37.5) | 1.17 (0.86 to 1.59)  1.24 (0.78 to 1.95) |
| Proportion of births attended by:  Named/partner midwife  Other team midwife | 0 if < 50%  0 if > 25% | 74 (44.1)  126 (75.0) | 109 (64.3)  144 (85.7) | 1.03 (0.83 to 1.29)  1.18 (0.88 to 1.57) |
| Proportion of postnatal visits by:  Named/partner midwife  Other team midwife | 0 if < 50%  0 if > 25% | 32 (19.1)  70 (41.6) | 78 (46.4)  102 (60.7) | 0.89 (0.65 to 1.22)  0.91 (0.72 to 1.14) |
| *Overall implementation fidelity (all measures)* | 0 if < 0.75 | 48 (28.6) | 87 (51.8) | 0.86 (0.66 to 1.13) |
| **Implementation acceptability measures** |  |  |  |  |
| Proportion of women who would prefer a POPPIE midwife to be the main person for their maternal care if they were to have another baby | 0 if < 75% | 3/89 (3.4) | 35/89 (39.3) | 1.06 (0.46 to 2.40) |
| **Implementation Composite Score** | 0 if < 0.75 | 15/89 (8.9) | 41/89 (46.1) | 0.82 (0.49 to 1.35) |

Data are n (%). n/N (%) indicates that the denominator only includes participants with a relevant measurement for that outcome. *Ranking score approach specified in Table S3. Abbreviations: CI, confidence interval.
