# Supplementary material for: A continuity of care programme for women at risk of preterm birth in the UK: Process evaluation of a hybrid randomised controlled pilot trial
Source: PLoS One. 2023 Jan 12;18(1):e0279695. doi: 10.1371/journal.pone.0279695 (PMC9836307; doi:10.1371/journal.pone.0279695)
Supplement: S4 Table — (DOCX) [file pone.0279695.s006.docx]

**S4 Table: Additional process outcomes for mothers and babies**

|  | **POPPIE**  **care**  **(n=168)** | **Standard**  **care**  **(n=163)** | **Effect size**  **(95% CI)** |
| --- | --- | --- | --- |
| Mean number of antenatal visits (any healthcare provider) | 11.15 (4.08) | 9.31 (3.45) | 1.84 (1.02 to 2.65) |
| Mean number of missing antenatal appointments | 0.37 (1.12) | 0.49 (0.88) | -0.12 (-0.34 to 0.10) |
| Mean number of obstetric clinic visits | 2.19 (1.62) | 2.07 (1.46) | 0.12 (-0.21 to 0.46) |
| Mean number of antenatal day unit attendances | 2.05 (2.46) | 2.50 (2.58) | -0.44 (-0.99 to 0.10) |
| Mean number of antenatal hospital inpatient nights | 0.95 (2.15) | 1.29 (2.41) | 0.34 (-0.84 to 0.15) |
| Mean number of postnatal inpatient nights | 1.80 (1.96) | 2.06 (1.87) | -0.26 (-0.67 to 0.16) |
| Mean number of postnatal visits | 6.64 (3.30) | 4.51 (10.63) | 2.13 (0.41 to 3.84) |
| Antenatal referrals  Smoking cessation  Mental health  Domestic violence (DV)  Pregnancy Plus  Safeguarding  *Mental health*  *DV*  *Mental health & DV*  *Social care / Housing*  *Substance misuse*  Other referrals  *Obstetrician*  *Fetal Medicine*  *Medical specialists*  *Consultant midwife*  *Diabetic midwife*  *Kaleidoscope midwife*  *Physiotherapist*  *Other*† | 151 (89.9)  41 (24.4)  12 (7.1)  3 (1.8)  12 (7.1)  20 (11.9)  7 (35)  4 (20)  1 (5)  5 (25)  3 (15)  139 (82.7)  101/139 (72.6)  3/139 (2.1)  14/139 (10.1)  2/139 (1.4)  6/139 (4.3)  4/139 (2.9)  7/139 (5.1)  3/139 (2.1) | 155 (95.1)  39 (23.9)  7 (4.3)  2 (1.2)  12 (7.4)  9 (5.5)  3 (33.3)  1 (11.1)  0 (0.00)  4 (44.4)  2 (22.2)  145 (89.0)  106/145 (73.1)  3/145 (2.1)  13/145 (8.9)  4/145 (2.7)  7/145 (4.8)  3/145 (2.1)  4/145 (2.7)  5/145 (3.4) | 0.95 (0.89 to 1.01)  1.02 (0.70 to 1.49)  1.66 (0.67 to 4.12)  1.46 (0.25 to 8.60)  0.97 (0.45 to 2.10)  2.16 (1.01 to 4.59)  0.93 (0.85 to 1.02) |
| Mean number of postnatal inpatient nights | 1.80 (1.96) | 2.06 (1.87) | -0.26 (-0.67 to 0.16) |
| Mean number of postnatal visits (midwife) | 6.64 (3.30) | 4.51 (10.63) | 2.13 (0.41 to 3.84) |
| Postnatal referrals  General Practitioner  Domestic Violence  Mental Health  Perineal clinic  Perinatal loss clinic  Neonatologist/Paediatrician  Neonatal jaundice clinic  Other‡ | 47 (28.0)  5/47  3/47  2/47  3/47  3/47  10/47  9/47  12/47 | 36 (22.1)  3/36  0/36  0/36  1/36  3/36  12/36  7/36  11/36 | 1.27 (0.87 to 1.85) |
| Maternal A&E attendance/ re-admission | 8 (4.8) | 14 (8.6) | 0.55 (0.24 to 1.29) |
| Neonatal A&E attendance/ re-admission | 22 (13.1) | 16 (9.8) | 1.33 (0.73 to 2.45) |
| Mean number of emergency attendances & admissions (mother and baby all cause) | 0.17 (0.48) | 0.83 (7.76) | -0.66 (-1.85 to 0.54) |

Data are n (%) or mean (standard deviation). DV: domestic violence; A&E: Accident and Emergency.

† Other antenatal referrals such as dietician service, genetic counselling or health visiting.

‡ Other postnatal referrals such as dermatology, ophthalmology, endocrinology or cardiology.
